# Towards a better registration of neuroendocrine neoplasms: The results of the Italian retrospective population-based study

**DOI:** 10.1177/03008916251317128

**Published:** 2025-02-26

**Authors:** Annalisa Trama, Francesco Cuccaro, Domenico Damiani, Alice Bernasconi, Francesco Panzuto, Nicola Fazio, Simona Carone, Maria Giovanna Burgio Lo Monaco, Rossella Bruni, Adele Caldarella, Annalisa Roselli, Barbara Cortini, Rosalba Amodio, Walter Mazzucco, Silvia Leite, Chiara Lupi, Maddalena Baracco, Eva Carpin, Antonella Dal Cin, Annarita Fiore, Laura Memo, Stefano Guzzinati, Antonietta Alfia Maria Torrisi, Antonina Torrisi, Margherita Ferrante, Maria Teresa Pesce, Alessandra Sessa, Antonietta Minichino, Nadia Di Pascale, Maria Francesca Vitale, Mario Fusco, Ilaria Girolami, Massimo Milione, Fabrizio Stracci

**Affiliations:** 1 Evaluative Epidemiology Unit, Fondazione IRCCS Istituto Nazionale dei Tumori (INT), Milan, Italy; 2 Puglia Cancer Registry, Section of Local Health Authority Barletta-Andria-Trani, Barletta, Italy; 3 Department of Pathology, Provincial Hospital of Bolzano (SABES-ASDAA), Lehrkrankenhaus der Paracelsus Medizinischen Privatuniversität, via Lorenz Böhler, 5, 39100 Bolzano-Bozen, Italy; 4 Department of Medical-Surgical Sciences and Translational Medicine, Digestive Disease Unit, Sant’ Andrea University Hospital, ENETS Center of Excellence, Sapienza University of Rome, Rome, Italy; 5 Division of Gastrointestinal Medical Oncology and Neuroendocrine Tumors, IRCCS European Institute of Oncology (IEO), Milan, Italy; 6 Puglia Cancer Registry, Section of Local Health Authority Taranto, Taranto, Italy; 7 Puglia Cancer Registry, Coordination Centre, Strategic Regional Agency for Health and Social Care - Puglia, Bari, Italy; 8 Tuscany Cancer Registry, Clinical Epidemiology Unit Institute for Cancer Research, Prevention and Clinical Network, Florence, Italy; 9 Clinical Epidemiology and Cancer Registry Unit, Azienda Ospedaliera Universitaria Policlinico, Palermo, Italy; 10 Cancer Registry of Umbria, Punto Zero, Perugia, Italy; 11 School of Public Health, Department of Medicine, University of Perugia, Perugia, Italy; 12 Veneto Tumour Registry, Epidemiological Department, Azienda Zero, Padova, Italy; 13 Cancer Registry Catania-Messina-Enna, G. Rodolico – San Marco Polyclinic University Hospital, Catania, Italy; 14 ASL Caserta - U.O.C. Monitoraggio rischio ambientale e Registro Tumori, Caserta, Italy; 15 Napoli 3 Sud Cancer Registry, Brusciano, Italy; 16 1st Pathology Division, Department of Pathology and Laboratory Medicine, Fondazione IRCCS Istituto Nazionale dei Tumori, Milan, Italy; 17 Public Health Section, Department of Medicine and Surgery, University of Perugia, Perugia, Italy

**Keywords:** population based cancer registries, neuroendocrine neoplasms, epidemiology

## Abstract

**Background:**

The increasing incidence, rapidly evolving classification, rarity and heterogeneity of neuroendocrine neoplasms (NENs) pose challenges to NEN registration including difficulty in distinguishing neuroendocrine carcinoma (NEC) and neuroendocrine tumours (NETs). Thus, in Italy a higher NEC incidence was reported. Focusing on gastroenteropancreatic (GEP) NEN, we aimed to review GEP NEN, and in particular cases of neuroendocrine carcinoma, not otherwise specified (NOS) and estimate the incidence of NEN, NET and NEC of the GEP.

**Methods:**

We launched a pilot study examining cases of neuroendocrine carcinomas NOS (ICD-O3 code 8246) of GEP incidents in the years 2012-2020. Cancer registries (CRs) reviewed information included in the pathology report regarding differentiation and tumour cells proliferation to decide whether to confirm the case as neuroendocrine carcinoma NOS or register it as NET or NEC. After the review, we estimated the GEP NEN, NET and NEC incidence.

**Results:**

Nine CRs contributed to the pilot study. After review, in all CRs, only 31% of GEP NOS neuroendocrine carcinomas were confirmed; 50% were recoded as NETs, and approximately 17% of cases were non-NENs. The IR of GEP NENs was 2.99/100,000, and the incidence of NETs was higher than that of NECs.

**Conclusion:**

After the review, the incidence of GEP NEN, NET and NEC in the eight Italian CRs involved was comparable to that reported in other European countries.

**Impact:**

Our results confirmed that heterogeneity of cancer registries in the registration of NEN requires collaborative work to define and promote a standard definition to be extended to all Italian registries.

## Introduction and objectives

Neuroendocrine neoplasms (NENs) include a heterogeneous group of tumours. The incidence rate of NENs is higher in the lung and varies from approximately 2/100,000 in the gastroenteropancreatic tract (GEP), to 0.2/100,000 in the skin and in the thyroid.^1^ Although NENs are rare cancers (incidence rate <6/100,000 in Europe), their incidence has increased over recent decades.^2,3^ This has been attributed to better disease knowledge, the spread of screening programmes, an improvement in diagnostic tools and other risk factors that are under discussion (e.g., family history, diet etc.).^2^

NENs receive little attention in current cancer statistics, which are mostly based on cancer topography. The RARECARE project proposed a list of rare cancers based on the combination of morphologies and topographies that allows us to reveal and identify NENs usually hidden in current statistics. Thus, the burden of NEN in terms of incidence, prevalence and survival has been published in Europe showing differences in incidence between European regions for both neuroendocrine tumours (NETs) and neuroendocrine carcinomas (NEC).^4^ NETs represent the well differentiated NENs whereas NECs are the poorly differentiated and, according to the current WHO classification,^5^ NETs can be divided into grade (G)1, G2 and G3 according to different cut-off of Ki-67 value, NECs are G3 by default.

In Italy, the results of the study of the Italian Association of population-based cancer registries (AIRTum)^6^ showed an incidence rate for GEP NEN in line with the European one but with an incidence for NECs higher than that of NETs. These data seem to contradict what has been published in the literature, which instead reports an opposite situation, namely a clear prevalence of well-differentiated forms compared to poorly differentiated ones.^7,8^ The rapidly evolving classification and rarity of NENs pose challenges to cancer registry registrars in recording NENs, including difficulty in distinguishing NETs and NECs. The high incidence of NEC may stem from cases of neuroendocrine carcinoma not otherwise specified (NOS), a morphology code which is included among NEC. In this context, it is important to disentangle heterogeneity due to delay in adequately reporting and registration errors from real problems in NEC diagnosis. To this aim, the AIRTUM has started a working group (WG) with the Italian Association of Neuroendocrine Tumours (Itanet). This collaboration, beside providing guideline for quality reporting and coding, has the scope of producing up to date quality data on NENs.

In this context, a pilot study, focused on GEP NENs, was conducted to 1) review GEP NENs, and in particular cases of NEC NOS, and 2) estimate the incidence of NENs, NETs and NECs of the GEP in the pilot project areas. The WG hypothesised that the high incidence of NECs may be due to the overuse of the neuroendocrine carcinoma NOS code. The lack of the specific SNOMED code in the pathology report and registrars’ limited experience in using information available in pathology reports to distinguish NET and NEC may have contributed to the unwarranted use of the nonspecific code.

Here we aim to report the results of this pilot study.

## Material and methods

### Selection of cases

Cases of GEP (International Classification of Disease for Oncology, 3rd revision ICD-O3] topographic codes: C15-C26) NENs (ICD-O3 morphology codes: 8013/3, 8002/3, 8041/3-8044/3, 8246/3, 8240/3 G1, 8249/3 G2 G3 G9, 8244/3, 8245/3, 8154/3, 8045/3, 8241/3, 8242/3, 8150/3, 8151/3, 8152/3, 8153/3, 8155/3, 8156/3, 8158/3) registered over the period 2012-2020 were included in the study. Cases of NEC NOS (ICD-O3 code 8246) were re-abstracted and recoded using available information and, mainly, pathology reports.

Nine Italian population-based cancer registries (CRs) (namely Alto Adige, Veneto, Toscana, Umbria, Puglia, Caserta and Napoli-3 Sud (Campania Region), Catania and Palermo (Sicilia Region) contributed to the pilot study. The pilot study population included about 17 million people (29% of Italian population) and is representative of national population based on participating CRs location.

### Revision of neuroendocrine carcinomas NOS

The AIRTUM-Itanet WG developed guidelines for the review of NEC NOS based on the experience of registrars and clinical experts of NEN. The guidelines recommended proceeding step by step: first consider the full text diagnosis written in the pathology report and code it according to a predefined list of ICD-O3 codes included in the guidelines; second, use the information available in the pathology report regarding tumour cell differentiation and proliferation (mitotic count and Ki-67) to distinguish NETs and NECs based on the recommendations included in the guidelines. These recommendations are summarised below.

- 8246/3 G3 (NEC) if the neoplasm is poorly differentiated, the mitotic index is > 20 mitoses/mm2/HPF and/or Ki67 > 20%;
- 8249/3 G3 (NET-G3) if the neoplasm is well differentiated, the mitotic index is > 20 mitoses/mm2/HPF and/or Ki67 > 20% (this nosographic entity is expected to be the rarest);
- 8249/3 G2 (NET-G2) if the neoplasm is well differentiated, the mitotic index is between 2 and 20 mitoses s/mm2/HPF and/or Ki67 is between 3 and 20%;
- 8240/3 G1 (NET-G1) if the neoplasm is well differentiated, the mitotic index is < 2 mitoses or rare mitoses /mm2/HPF and/or Ki67 is < 3%

Monthly meetings were organised between registrars and NENs clinical experts to discuss difficult-to-review cases. All doubtful cases were examined by at least two NEN expert pathologists and two experienced registrars who compared all available information (e.g. pathology reports, hospital discharge forms, death certificate, diagnosis reported in clinical documentation) to reach a final decision able at least to distinguish NETs from NECs.

The results of these discussions were used to add suggestions and clarifications to the recommendations included in the guidelines. The CRs examined the pathology reports based on the guidelines provided and decided whether to confirm the case as NEC NOS, including it among the NECs or to register it as NETs.

### Statistical analysis

Based on the results, the CRs estimated and shared the incident rate (IR) centrally by histology (NEC, NET, mixed NENs and other functioning and non-functioning NENs), age, sex and tumour site. CRs provided also age standardised (EU population 2013) IR. Each CR shared centrally the IRs only, not the incident cases nor the exact underlying population, thus it was not possible to calculate 95% confidence intervals. To calculate the IRs of NENs, NETs and NECs of the GEP in the pilot we made a weighted average of the IRs provided by the CRs, using as weights the population living in each area (province or region) according to National Statistical Institute (ISTAT) 2013 populations.

### Data statement

We analysed aggregated data provided by the CRs contributing to the pilot study. We do not have individual data. Aggregated level data, in the form of rates, can be only shared after express permission from the participating registries. These data should be requested by contacting the corresponding author.

## Results

Table 1 reports the results of the review. A total of 1325 cases of NEC NOS were examined, and only 31.3% were confirmed as NOS. 51.3% were changed to NETs (ICD-O3 morphology code: 8240/3 (27.8%); 8249 G2/3 (21.4%); 8249 G3/3 (2.0%)). Approximately 200 cases (17.4%) were excluded because they were found to be non-NEN (mainly adenocarcinomas with neuroendocrine aspects/differentiation, ICD-O3 morphology code: 8574/3).

**Table 1.** Number (N) of neuroendocrine carcinoma not otherwise specified (8246) reviewed together with the number modified, excluded and confirmed not specified carcinoma by year of diagnosis.

year of diagnosis	8246 Reviewed (N)	Modified (N)			Excluded (N)	8246 Confirmed (N)
		8240	8249 G2	8249 G3	No NENs^§^	
2012	119	41	35	0	12	31
2013	164	41	37	4	37	45
2014	146	42	29	3	25	47
2015	181	45	49	3	33	52
2016	189	43	41	3	34	68
2017	167	49	25	4	26	63
2018	190	59	40	6	30	56
2019	163	49	27	4	31	50
2020*	6	0	1	0	2	3
Total	1325	369	284	27	230	415

* Only contribution to the review from Napoli CR.

§ NEN=neuroendocrine neoplasm

The IR of GEP NENs was 2.99/100,000, the age-standardized IR was 3.2/100,000. The IR of NETs was three times higher than that of NECs, and NET-G1s were the most common NETs. For all NEN morphologies, the IR was slightly higher in males than females and was higher (approximately 8/100,000) for those older than 65 years (Table 2).

**Table 2.** Crude and age standardised (ASR) incidence rate of neuroendocrine neoplasms (NENs) by sex and age group. Rates are x 100,000.

	Crude rate	ASR	Sex		Age group					
			Male	Female	0-14	15-44	45-54	55-64	65-74	75+
NECs (ICD-O3 code 8013/3, 8002/3, 8041/3-8044/3, 8246/3)	0.69	0.68	0.77	0.56	0.00	0.07	0.52	1.20	2.09	2.31
NETs (ICD-O3 code 8240/3G1, 8249/3 G2, G3, G9)	2.16	2.42	2.22	2.07	0.43	1.25	2.35	3.46	5.58	5.28
Mixed MANEC/MINEN (ICD-O3 code 8244/3, 8245/3, 8154/3, 8045/3)	0.11	0.12	0.14	0.10	0.00	0.00	0.13	0.14	0.31	0.49
Other functioning and non functioning (ICD-O3 code 8241/3, 8242/3, 8243/3, 8150/3, 8151/3, 8152/3, 8153/3, 8155/3, 8156/3, 8158/3)	0.02	0.03	0.03	0.03	0.01	0.01	0.03	0.02	0.08	0.08
NENs	2.99	3.24	3.16	2.76	0.44	1.33	3.03	4.82	8.07	8.16

NEC: neuroendocrine carcinomas; NET: neuroendocrine tumours; ICD-O3: International Classification of Disease for Oncology, 3rd revision.

IR by site differed between NETs and NECs. Most common sites of NECs were pancreas and colon followed by stomach. Most common sites of NETs were pancreas and small intestine followed by colon, stomach and rectum (Table 3).

**Table 3.** Incidence rates of neuroendocrine neoplasms (NENs) by major sites of the gastroenteropancreatic tract (GEP). Rates are x 100,000.

	GEP sites					
	Esophagus	Stomach	Small intestine	Colon	Rectus	Pancreas
NECs	0.04	0.11	0.03	0.12	0.04	0.17
NETs	0.01	0.28	0.57	0.38	0.15	0.62
Mixed MANEC/MINEN	0.00	0.02	0.00	0.04	0.01	0.02
Other functioning and non functioning	0.00	0.00	0.00	0.01	0.00	0.02
NENs	0.04	0.41	0.61	0.55	0.20	0.82

NEC: neuroendocrine carcinomas; NET: neuroendocrine tumours.

After the revision the proportion of NEC NOS was around 15% of all GEP NEN GEP.

## Discussion

Our results confirmed that the high IR of GEP NECs reported by the AIRTUM study^6^ was mainly due to misclassification of NEC NOS. After the review the IR of NETs resulted higher than that of NECs, correcting and updating the IR of the previous publication which reported an IR too high for NEC.^6^

This study highlighted that NENs can be misdiagnosed by registrars as well as by non-expert pathologists. Notably we had 17% of cases which were not even NENs. In recording NEN, registrars may encounter several problems: by consulting the different data sources, they may find pathological reports with obsolete (e.g. carcinomas with neuroendocrine differentiation) or incomplete description (e.g. neuroendocrine neoplasia without information on differentiation or proliferative status) or with discordant indications (e.g. between Ki-67 and the mitotic index); conflicting information may also be found between the biopsy report and the surgical specimen report, or between primary and metastatic sites (for example, a NEN may be well differentiated in one source and poorly differentiated in the other)^9-11^; for a very small percentage of cases registrars find the morphologic diagnosis of NEN as written down in medical records, death certificates or other clinical documentation, without the availability of the actual pathology report. In these cases, a registrar may end up registering a NEN as NOS. During the pilot study, the multidisciplinary consensus approach made it possible to reach a shared and reproducible decision.

NENs are rare and heterogeneous malignancies and therefore difficult to diagnose especially by non-NEN-dedicated pathologists and outside of a NEN-referral centre. Our results showed that misclassification due to CR can be reduced or even solved by clear recommendations and promoting discussions involving both clinical experts and registrars. However, the guidelines and training are based on the pathology report, therefore, if the latter does not include the relevant information recommended by the European Society of Neuroendocrine Tumours (ENETS)^12^ for recording pure NENs and distinguishing between NETs and NECs, it is impossible for CRs to adequately register these neoplasms. During the monthly meeting discussions, the registrars documented the poor quality of some of the pathological reports, but with an overall improvement over more recent year. In the past, the ENETS introduced standardised reporting for NENs in both endoscopic^13^ and pathological reports.^12^ This initiative significantly enhanced clinical management and research by ensuring clear, uniform communication of crucial data. It facilitated accurate diagnoses, enabled consistent treatment planning, and improved monitoring and research collaboration. The primary utility of standardised reporting lies in its role in advancing patient outcomes and deepening our understanding of NENs through more effective and coordinated healthcare practices. Such approaches should be supported to minimise ambiguity in communicating tumour characteristics.

The IRs of GEP NETs and NECs provided by this study were comparable to those reported in other European countries (in Norway NEC IR was 0.4/100,00^14^; in Switzerland ASR for GEP NEN was about 4/100,000 and NEC IR was 0.9/100,000 and 0.5/100,000 in males and females respectively^15^; in the Netherlands NEC IR was 0,5/100,000^16^) but lower than those reported in the USA^17^ and Japan.^18^ Differences may be due to the different diagnostic pressure and availability of screening for GEP cancers which may increase the chance of identifying incidentalomas. However, the data are difficult to compare due to the limited literature distinguishing NETs and NECs, the different definitions of NENs, NETs and NECs, the different behaviour of NENs used in the studies, the different diagnosis periods, and the different GEP locations considered.^14,19-22^ Moreover, the introduction of a novel subtype NET G3 in 2019 played a role in pursuing a greater heterogeneity in registration procedures, leading to some abrupt switches in incidences, and consequent declining trend among NEC.^23^

Our study supports the importance of collaborative working to reduce the heterogeneity of NEN registration among CRs. Furthermore, it highlights the importance of defining and promoting a standard definition of NENs, NETs and NECs to be used for reporting epidemiological indicators in order to increase the comparability and quality of data within and between countries. Moreover, it is expected that reliable data, although outside the context of the CRs, may come from the Itanet database project, which has been collecting prospective data on new diagnoses of GEP NENs in Italy since 2019. It is likely to be able to provide useful information to enrich the epidemiological significance and the presentation modes of these rare neoplasms.^24^

Furthermore, this work confirms that the correct diagnosis of NENs can be difficult. Therefore, referral of patients with NENs should be strengthened within a network of expert centers. This would facilitate histological review even of cases diagnosed in centers with limited experience on these pathologies. Pathological review can, in fact, change the grade of the tumour in a significant percentage, up to a quarter, of cases.^25^

We are aware of the limitations of our study which is retrospective in nature, focused on a limited number of CRs, although representative of the Italian territory, a specific site (e.g. GEP) and a single histology (ICD-O3 morphological code 8246). On the other hand, a potential strength of our study is its population-based nature. Given the rarity of NENs, the epidemiology of these tumours is best studied in large population-based CRs. Another strong point is the review of updated cases and the close collaboration between registrars and NET experts (pathologists and clinicians) under the aegis of the relevant scientific societies, AIRTUM and Itanet respectively.

In Italy, the AIRTUM-Itanet WG proposed to expand the pilot study to all Italian CRs in order to provide reliable indicators for GEP NENs and the different types of GEP NENs (NETs, NECs, etc.). The national study which will include the review of cases and the definition of the IR will be an opportunity to define recommendations for CRs regarding the registration of the GEP NENs and to promote them in training courses and dissemination events. These activities will pave the way for a new way of recording NENs at national and international level and therefore for reliable and comparable epidemiological indicators across continents. To this extent we will leverage the upcoming European joint action on CRs (JA CancerWatch) to promote the Italian experience as a good practice to address quality problems of registration of NENs and ensure epidemiological data comparability.
